# Clinical utility of the lactate-to-albumin ratio for predicting mortality in elderly severe acute pancreatitis

**DOI:** 10.3389/fmed.2026.1754871

**Published:** 2026-05-04

**Authors:** Qingcheng Zhu, Yingtong Sun, Lingli Wu, Huihui Wang, Yueli Chen

**Affiliations:** 1 Department of Emergency Medicine, Northern Jiangsu People’s Hospital Affiliated to Yangzhou University, Yangzhou, China; 2 Department of Emergency Medicine, Gaoyou People’s Hospital, Gaoyou, China

**Keywords:** elderly, lactate-to-albumin ratio, LAR, mortality, severe acute pancreatitis

## Abstract

**Background:**

Elderly patients with severe acute pancreatitis (SAP) have high mortality, yet reliable early risk stratification tools remain limited. The lactate-to-albumin ratio (LAR) has emerged as a potential prognostic biomarker. This study aimed to evaluate the association between LAR and in-hospital mortality in elderly SAP patients.

**Methods:**

We retrospectively analyzed 313 patients aged ≥65 years with SAP admitted to the intensive care unit of a tertiary teaching hospital between 2016 and 2024. Demographic, clinical, and laboratory data were collected at admission. LAR was calculated from arterial lactate and serum albumin. Cox proportional hazards models were used to identify independent predictors of in-hospital mortality. Predictive performance of LAR was assessed using receiver operating characteristic (ROC) curves, Kaplan–Meier survival analysis, and threshold effect modeling.

**Results:**

Ninety-four (30.0%) patients died during hospitalization. Non-survivors had significantly higher LAR values than survivors. Multivariable Cox regression confirmed LAR as an independent predictor of all-cause in-hospital mortality (HR 1.50, 95% CI 1.27–1.78; *p* < 0.001). The optimal LAR cutoff of 0.79 identified patients at high risk of death. ROC analysis showed that LAR (AUC 0.700) performed better than albumin (AUC 0.432) and was comparable to lactate (AUC 0.688) and SOFA score (AUC 0.732). Kaplan–Meier curves demonstrated significantly reduced survival in the high-LAR group.

**Conclusion:**

LAR is an independent predictor of all-cause in-hospital mortality in elderly SAP patients, offering a simple and effective biomarker for early risk stratification and clinical decision-making in this high-risk population.

## 1 Introduction

Acute pancreatitis (AP) is a pancreatic inflammatory disorder with clinical manifestations ranging from mild, self-limiting episodes to severe, life-threatening disease (1). Its global incidence is estimated at 30–40 per 100,000 annually and is rising among elderly populations due to biliary pathology and comorbid conditions (2). While the majority of patients experience mild disease, 20–25% progress to severe acute pancreatitis (SAP), characterized by persistent organ failure and local complications (3). Mortality in SAP can reach 50%, particularly in older or medically fragile patients (4).

Advanced age is a recognized risk factor for poor outcomes in AP, primarily due to diminished organ reserve, a higher burden of comorbidities, and chronic systemic inflammation (5). Elderly patients with SAP are particularly susceptible to multisystem organ failure and elevated in-hospital mortality (6). Zhu *et al*. reported a 20.25% mortality rate among SAP patients aged ≥65 years, highlighting age as an independent prognostic factor (7). Consistently, data from a large intensive care unit (ICU) cohort indicated that patients over 60 experienced significantly worse outcomes and prolonged hospital stays compared with younger individuals (8). These findings underscore the importance of early identification of high-risk elderly patients and the implementation of tailored management strategies to improve survival.

Several scoring systems, including the Ranson criteria, the Acute Physiology and Chronic Health Evaluation II (APACHE II), and the Sequential Organ Failure Assessment (SOFA), are commonly employed to evaluate the severity of AP (9). Although these tools integrate multiple clinical and laboratory parameters to provide a comprehensive assessment, their complexity often requires extensive data collection, potentially delaying timely clinical decision-making (10). In emergency settings, such delays may impede early intervention and contribute to worse outcomes (11). Therefore, there is an increasing demand for a simple, readily available, and reliable biomarker capable of rapidly predicting disease severity in AP patients.

Lactate serves as an important marker of tissue hypoperfusion and metabolic stress and has been increasingly studied for its prognostic significance in SAP (12). Evidence indicates that lactate clearance independently predicts mortality in SAP patients (13). However, elevated lactate can also arise from sepsis, systemic inflammation, or certain medications (14), necessitating careful interpretation. Albumin, a negative acute-phase protein, reflects inflammatory burden and correlates with disease severity and patient outcomes (15). Its levels, however, may be influenced by nutritional status or chronic inflammation, limiting its reliability as an isolated prognostic indicator (16).

The lactate-to-albumin ratio (LAR) represents the interplay between lactate production and albumin status (17). High LAR has been associated with adverse outcomes across various critical conditions, including sepsis, traumatic brain injury, cardiac arrest, and acute respiratory failure (18, 19). Despite these findings, its prognostic value in elderly patients with SAP remains unclear. This study therefore aimed to evaluate the association between LAR and in-hospital mortality in this high-risk population, providing evidence to enhance early risk stratification and inform individualized management strategies.

## 2 Materials and methods

### 2.1 Study design and patient selection

This retrospective investigation was performed in the ICU comprising 78 beds at a tertiary hospital in Jiangsu Province, China. The study protocol received approval from the Ethics Review Board of Northern Jiangsu People’s Hospital (approval No. 20250328) and was implemented in accordance with the ethical standards of the revised Declaration of Helsinki. Given the retrospective nature of the research, the requirement for informed consent was formally waived.

Between January 2016 and December 2024, patients diagnosed with SAP were retrospectively screened. AP was identified according to the revised Atlanta Classification (2012), requiring at least two of the following features: (1) abdominal pain characteristic of pancreatitis, (2) elevation of serum amylase or lipase to more than three times the normal upper limit, or (3) radiological evidence consistent with AP (20). Severe disease was characterized by persistent organ dysfunction lasting over 48 h, reflected by a modified Marshall score of ≥2 in at least one system, including respiratory, cardiovascular, or renal (20).

Eligible participants were those aged 65 years or above who fulfilled the diagnostic criteria for SAP and had complete demographic, clinical, and laboratory information collected within the first 24 h after ICU admission. Patients were excluded if they had a history of malignancy, prior pancreatic surgery, abdominal trauma, or decompensated liver cirrhosis. In addition, patients transferred from other hospitals were excluded to ensure that laboratory measurements reflected the initial hospital presentation. Patients with missing data in variables required for the analysis were excluded, and a complete-case approach was applied. The overall proportion of missing data was low (<3% across all variables), suggesting minimal risk of bias related to missingness. Following eligibility assessment, all included individuals were classified into survival and non-survival groups based on their in-hospital outcomes.

### 2.2 Data collection

Data were retrieved from the electronic medical record system, encompassing demographic characteristics, medical history, and vital signs at admission, including respiratory rate (RR), heart rate (HR), systolic blood pressure (SBP), and diastolic blood pressure (DBP). Body mass index (BMI) and SOFA scores were assessed upon admission.

Laboratory assessments included hematologic profiles (counts of red and white blood cells, platelets, hemoglobin, and hematocrit), biochemical indicators of hepatic function (total bilirubin, alanine and aspartate aminotransferases, and albumin), renal and metabolic parameters (electrolytes and glucose), and coagulation indices (prothrombin time (PT), activated partial thromboplastin time (APTT), and international normalized ratio (INR)). In addition, pancreatic enzymes (amylase, lipase) and arterial blood gas results—pH, arterial partial pressure of oxygen (PaO₂), arterial partial pressure of carbon dioxide (PaCO₂), and lactate—were reviewed. In this study, “admission” was defined as ICU admission, and all clinical and laboratory variables were collected within the first 24 h following ICU admission. The requirement for vasopressor administration at ICU admission was documented, and the LAR was calculated accordingly. LAR was computed as arterial lactate (mmol/L) divided by serum albumin (g/dL).

### 2.3 Primary outcome

The primary outcome was all-cause in-hospital mortality among elderly patients with SAP. Secondary outcomes included 28-day and 90-day all-cause mortality, defined as death occurring within 28 or 90 days of hospital admission, respectively. Mortality status was obtained from hospital electronic records, and no post-discharge follow-up was required.

### 2.4 Statistical analysis

The distribution of continuous variables was evaluated with the Kolmogorov–Smirnov test. Normally distributed data were summarized as mean ± standard deviation, while non-normally distributed data were expressed as median and interquartile range. Differences between groups were examined using either the Student’s t-test or the Mann–Whitney U test for continuous variables, and the chi-square or Fisher’s exact test for categorical comparisons.

The relationship between candidate variables and hospital mortality was analyzed in two stages. First, univariable Cox proportional hazards models were applied to identify factors associated with outcomes. Those reaching statistical significance (*p* < 0.05) were subsequently included in a multivariable Cox regression model to determine independent prognostic factors. Results are presented as hazard ratios (HRs) with corresponding 95% confidence intervals (CIs). The proportional hazards assumption was evaluated using Schoenfeld residuals. Multicollinearity among variables was assessed using variance inflation factors (VIFs).

Discriminative performance of lactate, albumin, LAR, and SOFA scores for predicting mortality was evaluated through receiver operating characteristic (ROC) analysis. The area under the curve (AUC), sensitivity, and specificity were calculated for each index, and the optimal LAR cut-off value was identified using Youden’s index. Based on this threshold, patients were categorized into low- and high-LAR groups, and survival differences were visualized by Kaplan–Meier curves with comparisons made using the log-rank test. The clinical relevance of the LAR was evaluated through decision-curve analysis (DCA).

Potential non-linear associations between LAR and mortality were further explored using smoothed curve fitting and segmented regression analysis. A two-piecewise linear model was employed to determine possible inflection points, selected according to the highest model likelihood.

All statistical analyses were performed using R (version 3.6) and SPSS (version 24). Two-tailed *p* values < 0.05 were regarded as statistically significant.

## 3 Results

### 3.1 Baseline characteristics

**Figure 1:**
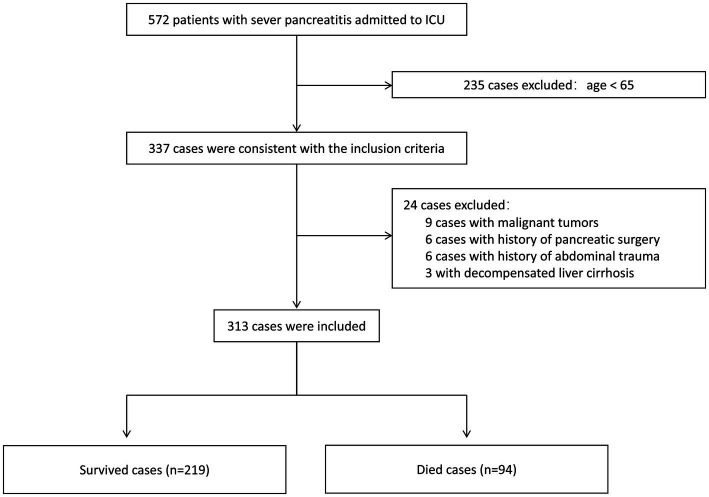
Flow chart of patient enrollment. ICU, intensive care unit.

A total of 337 elderly patients with SAP were initially screened, with 24 patients excluded based on predetermined criteria. Ultimately, 313 patients were included in the study, comprising 219 in the survival group and 94 in the non-survival group (Figure 1).

Baseline characteristics at ICU admission are summarized in Table 1. Compared with survivors, non-survivors were significantly older and exhibited higher RR and HR, but lower SBP and DBP. SOFA scores on admission were also markedly higher in non-survivors (10 (7–12) vs. 6 (4–8), *p* < 0.001). Regarding laboratory findings, non-survivors had significantly higher creatinine, urea nitrogen, INR, PT, APTT, lactate, and LAR. Albumin and calcium levels were significantly lower in the non-survival group. Clinically, vasopressor use occurred more frequently in non-survivors (81.9% vs. 43.4%, *p* < 0.001). No significant differences were found in sex distribution, BMI, or the prevalence of major comorbidities between groups.

**Table 1:** Baseline characteristics of selected patients.

Characteristics	Survived (*n* = 219)	Died (*n* = 94)	*p*
Male, *n* (%)	127 (58.0)	50 (53.2)	0.509
Age, years	74 (68–80)	78 (73–84)	0.002
Height (cm)	168 (165–170)	168 (161–173)	0.239
Weight (kg)	80.0 (68.1–90.3)	80.2 (71.9–95.8)	0.371
BMI (kg/m^2^)	28.3(24.3–31.9)	28.6 (25.8–34.0)	0.193
Comorbidities, *n* (%)			
COPD	16 (7.3)	2 (2.1)	0.124
Coronary artery disease	62 (28.3)	22(23.4)	0.448
Hypertension	77 (35.2)	39 (41.5)	0.350
Diabetes mellitus	71 (32.4)	35 (37.2)	0.487
Vital signs			
Respiratory rate (/min)	24 (19–29)	28 (22–34)	0.001
Heart rate (/min)	100 (86–118)	112 (90–129)	0.015
Systolic blood pressure (mmHg)	97 (89–116)	95 (77–106)	0.008
Diastolic blood pressure (mmHg)	47 (44–56)	46 (38–54)	0.013
SOFA	6 (4–8)	10 (7–12)	<0.001
Laboratory data			
Red blood cell (*10^12^/L)	3.8 (3.3–4.4)	3.6 (3.2–4.4)	0.560
White blood cell (*10^9^/L)	11.7 (8.3–17.3)	14.70 (8.5–18.8)	0.103
Platelet (*10^9^/L)	197 (157–252)	219 (142–291)	0.651
Neutrophil (%)	83.0 (76.2–87.0)	83.0 (68.9–88.9)	0.737
Lymphocyte (%)	7.2 (5.0–12.0)	7.0 (4.0–10.1)	0.107
Hemoglobin (g/L)	113 (100–132)	111 (100–130)	0.549
Hematocrit (%)	34.2 (30.0–39.9)	34.6 (30.7–40.0)	0.760
Total bilirubin (mg/dL)	1.1 (0.6–1.9)	1.0 (0.6–3.5)	0.254
Alanine aminotransferase (U/L)	44 (21–160)	44 (23–112)	0.761
Aspartate aminotransferase (U/L)	55 (31–173)	71 (32–128)	0.620
Lactate dehydrogenase (U/L)	358 (256–501)	357 (244–531)	0.943
Creatinine (mg/dL)	1.3 (0.9–1.9)	1.5(1.1–2.2)	0.022
Urea nitrogen (mg/dL)	25 (18–43)	36 (21–60)	0.003
Albumin (g/dL)	2.9 (2.5–3.5)	2.9 (2.2–3.3)	0.049
Sodium (mmol/L)	139 (136–141)	138 (135–142)	0.549
Potassium (mmol/L)	4.1 (3.8–4.5)	4.2 (3.8–4.8)	0.108
Chloride(mmol/L)	104 (101–109)	106 (100–111)	0.491
Calcium (mmol/L)	2.08 (1.88–2.22)	2.03 (1.85–2.13)	0.041
Glucose (mmol/L)	7.5 (5.8–10.5)	7.6 (6.2–9.8)	0.834
Amylase (U/L)	166 (69–314)	180 (69–517)	0.258
Lipase (U/L)	168 (49–731)	171 (53–967)	0.455
International normalized ratio	1.3 (1.1–1.6)	1.5 (1.2–1.8)	0.048
Prothrombin time (seconds)	14.4 (13.0–16.2)	15.3 (13.5–18.5)	0.015
Activated partial thromboplastin time (seconds)	30.4 (26.0–35.1)	32.1 (27.3–39.3)	0.039
Arterial pH	7.35 (7.26–7.42)	7.32 (7.18–7.38)	0.004
PaO_2_ (mmHg)	91 (69–130)	85 (75–114)	0.818
PaCO_2_ (mmHg)	40 (34–47)	39 (32–46)	0.522
Lactate (mmol/L)	1.9 (1.3–2.9)	3.0 (1.8–5.3)	<0.001
LAR	0.66 (0.43–1.00)	1.05 (0.67–2.36)	<0.001
Vasopressor, *n* (%)	95 (43.4)	77 (81.9)	<0.001

BMI, Body mass index; COPD, Chronic obstructive pulmonary disease; SOFA, Sequential Organ Failure Assessment; PaO_2,_ Partial pressure of arterial oxygen; PaCO_2_, Partial pressure of arterial carbon dioxide; LAR, Lactate-to-albumin ratio.

### 3.2 The LAR was an independent risk factor for mortality

Covariates with significant differences in Table 1 (*p* < 0.05) were included in the univariate Cox regression analysis. In this analysis, advanced age, lower SBP and DBP, higher SOFA score, elevated urea nitrogen, lower arterial pH, higher lactate levels, increased LAR, and vasopressor use were all significantly associated with all-cause in-hospital mortality (Table 2). Notably, the unadjusted LAR demonstrated a strong association with mortality (HR, 1.45; 95% CI, 1.28–1.65; *p* < 0.001).

**Table 2:** Univariate Cox regression analysis of variables for all-cause in-hospital mortality.

Variables	Hospital mortality		
	HR	95% CI	*P*
Age	1.06	1.03–1.08	<0.001
Respiratory rate	1.02	0.99–1.04	0.083
Heart rate	1.01	1.00–1.02	0.114
Systolic blood pressure	0.99	0.97–1.00	0.043
Diastolic blood pressure	0.98	0.97–0.99	0.021
SOFA	1.15	1.09–1.21	<0.001
Creatinine	1.12	0.99–1.26	0.066
Urea nitrogen	1.01	1.00–1.02	0.007
Albumin	0.79	0.59–1.05	0.104
Calcium	0.84	0.45–1.56	0.578
International normalized ratio	0.98	0.89–1.07	0.619
Prothrombin time	1.00	0.98–1.02	0.855
Activated partial thromboplastin time	1.00	0.99–1.01	0.914
Arterial pH	0.08	0.02–0.37	0.001
Lactate	1.16	1.10–1.22	<0.001
LAR	1.45	1.28–1.65	<0.001
Vasopressor	2.83	1.67–4.80	<0.001

HR, Hazard ratio; CI, Confidence interval; SOFA, Sequential Organ Failure Assessment; LAR, Lactate-to-albumin ratio.

In the adjusted Model I, which controlled for age, SBP, and DBP, LAR remained an independent predictor of in-hospital mortality (HR, 1.67; 95% CI, 1.45–1.91; *p* < 0.001). In the adjusted Model II, after additional adjustment for urea nitrogen, arterial pH, and vasopressor use, LAR continued to show a significant association with mortality (HR, 1.56; 95% CI, 1.33–1.85; *p* < 0.001). In the fully adjusted Model III, which further included SOFA score, the association between LAR and mortality remained statistically significant (HR, 1.50; 95% CI, 1.27–1.78; *p* < 0.001) (Table 3).

**Table 3:** Multivariate COX analysis of risk factors for in-hospital mortality.

Variables	Non-adjusted		Adjust I		Adjust II		Adjust III	
	HR (95% CI)	*p*	HR (95% CI)	*p*	HR (95% CI)	*p*	HR (95% CI)	*p*
LAR	1.45 (1.28–1.65)	<0.001	1.67 (1.45–1.91)	<0.001	1.56 (1.33–1.85)	<0.001	1.50 (1.27–1.78)	<0.001
LAR-group								
Low	Reference		Reference		Reference		Reference	
High	2.74 (1.77–4.23)	<0.001	2.85 (1.83–4.43)	<0.001	2.49 (1.58–3.91)	<0.001	2.18 (1.37–3.49)	0.001

Non-adjusted model adjusted for: None; Adjust I model adjusted for: Age, systolic blood pressure and diastolic blood pressure; Adjust II model adjusted for: Age, systolic blood pressure, diastolic blood pressure, urea nitrogen, arterial pH and the use of vasopressor. Adjust III model adjusted for: Age, systolic blood pressure, diastolic blood pressure, SOFA, urea nitrogen, arterial pH and the use of vasopressor. HR, Hazard ratio; CI, Confidence interval; LAR, Lactate-to-albumin ratio; SOFA, Sequential Organ Failure Assessment.

When stratified by the optimal LAR cut-off value of 0.79, determined using the Youden index, patients in the high LAR group had significantly higher mortality than those in the low LAR group. The corresponding HRs were 2.74 (95% CI, 1.77–4.23; *p* < 0.001) in the unadjusted Model, 2.85 (95% CI, 1.83–4.43; *p* < 0.001) in Model I, 2.49 (95% CI, 1.58–3.91; *p* < 0.001) in Model II and 2.18 (95% CI, 1.37–3.49; *p* = 0.001) in Model III (Table 3).

The proportional hazards assumption was tested using Schoenfeld residuals, and no significant violations were observed for LAR or any covariates included in the multivariable models (all *p* values > 0.05). Multicollinearity diagnostics showed no significant collinearity among LAR, SOFA score, lactate, or other covariates, with all VIF values below 3.

### 3.3 ROC curve analysis

**Figure 2:**
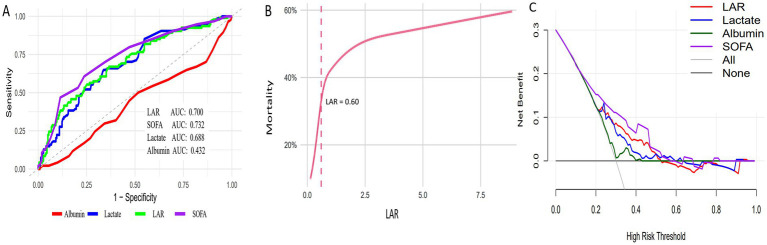
Association between LAR and in-hospital mortality of elderly patients with SAP. **(A)** ROC curves comparing the predictive performance of LAR, lactate, albumin, and SOFA score. **(B)** The non-linear association between LAR and in-hospital mortality, adjusted for covariates included in Model III (age, systolic blood pressure, diastolic blood pressure, urea nitrogen, arterial pH, SOFA score, and vasopressor use). **(C)** Decision curve analysis comparing the clinical net benefit of LAR, lactate, albumin, and SOFA score. LAR, lactate-to-albumin ratio; SAP, severe acute pancreatitis; ROC, receiver operating characteristic.

We constructed ROC curves for four indicators—LAR, lactate, albumin, and SOFA—to evaluate their ability to predict all-cause in-hospital mortality in elderly SAP patients (Figure 2A; Table 4). The AUCs were 0.700 (95% CI, 0.633–0.761) for LAR, 0.688 (95% CI, 0.626–0.752) for lactate, 0.432 (95% CI, 0.361–0.502) for albumin, and 0.732 (95% CI, 0.672–0.794) for SOFA. Pairwise comparisons demonstrated that the AUC of LAR was significantly higher than that of albumin (*p* < 0.001), whereas no significant differences were observed between LAR and lactate or between LAR and SOFA.

**Table 4:** Information of ROC curves in Figure 2A.

Variables	Survived	Died	AUC (95% CI)	Specificity	Sensitivity	Youden’s index
LAR	219	94	0.700 (0.633–0.761)	0.635	0.670	0.305
Lactate	219	94	0.688 (0.626–0.752)	0.662	0.649	0.311
Albumin	219	94	0.432 (0.361–0.502)	0.868	0.298	0.165
SOFA	219	94	0.732 (0.672–0.794)	0.763	0.606	0.369

ROC, The receiver operating characteristic; AUC, The area under the receiver operating characteristic curve; LAR, Lactate-to-albumin ratio; SOFA, Sequential Organ Failure Assessment.

### 3.4 The analysis of the non-linear association and clinical utility

After adjustment for covariates included in Model III, the association between LAR and hospital mortality was found to be non-linear (Figure 2B). Threshold effect analysis identified a significant inflection point at LAR = 0.60 (Table 5). A total of 122 patients had LAR ≤ 0.60, while 191 patients had LAR > 0.60. For LAR values ≤0.60, each 1-unit increase in LAR was associated with a markedly higher risk of hospital mortality (HR = 9.31, 95% CI 1.28–15.81; *p* = 0.012). In contrast, for LAR values >0.60, the association remained but was weaker (HR = 1.33, 95% CI 1.12–1.57; *p* = 0.043). The log-likelihood ratio test yielded a *p*-value of 0.035, indicating that the two-piecewise linear regression model provided a significantly better fit than the standard linear model.

**Table 5:** Information of nonlinear associations between LAR and mortality in Figure 2B.

Outcome	Hospital mortality	
	HR (95% CI)	*p*
Model I: Fitting model by standard linear regression	1.52(1.28–1.80)	<0.001
Model II: Fitting model by two-piecewise linear regression		
Inflection point	0.60	
≤ 0.60 (*n* = 122)	9.31 (1.28–15.81)	0.012
> 0.60 (*n* = 191)	1.33 (1.12–1.57)	0.043
*p* for log likelihood ratio test		0.035

LAR, Lactate-to-albumin ratio; HR, Hazard ratio; CI, Confidence interval.

To further assess clinical utility, DCA was conducted. LAR provided a greater net benefit than lactate and albumin across a range of threshold probabilities; however, the SOFA score consistently demonstrated the highest net benefit. (Figure 2C).

### 3.5 Kaplan–Meier curve analysis

**Figure 3:**
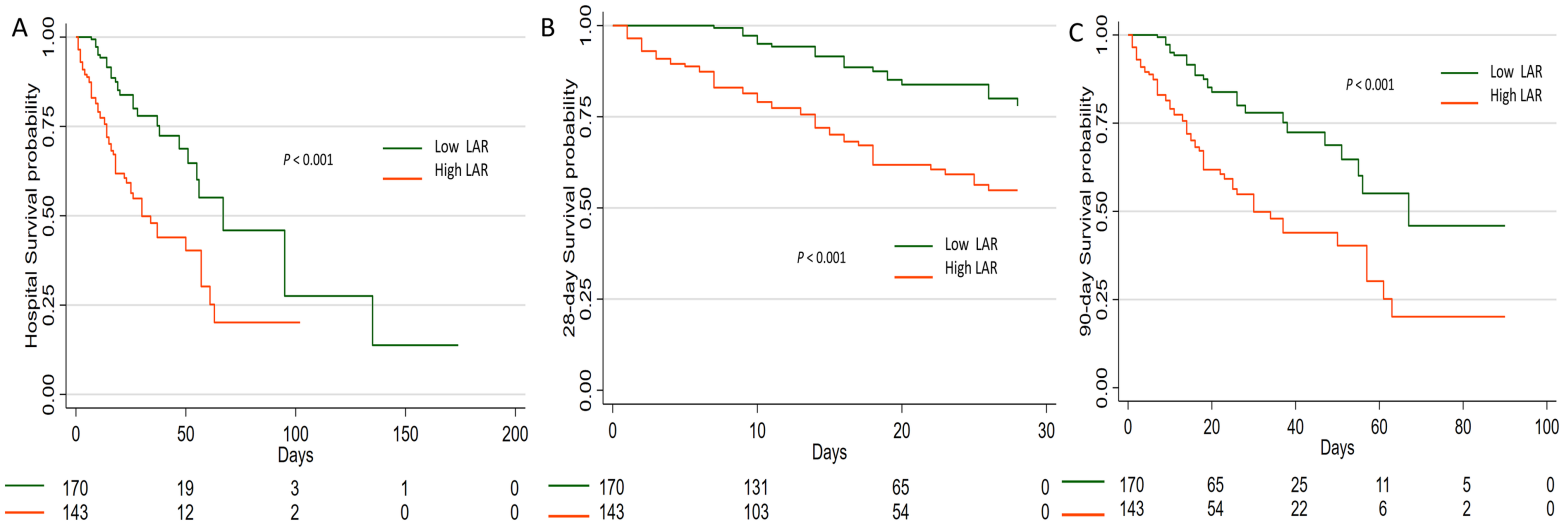
Kaplan-Meier survival curves for elderly patients with severe acute pancreatitis stratified by Lactate-to-Albumin Ratio (LAR). Patients were divided into a low-LAR group (<0.79) and a high-LAR group (≥0.79) based on the optimal cutoff determined by the Youden index. **(A)** In-hospital survival, **(B)** 28-day survival, and **(C)** 90-day survival. LAR, Lactate-to-Albumin Ratio.

Patients were stratified into two groups based on the optimal LAR cutoff identified by the Youden index: low LAR (<0.79) and high LAR (≥0.79). Kaplan–Meier survival analysis demonstrated significantly lower in-hospital survival in the high-LAR group compared with the low-LAR group. Consistently, 28-day and 90-day survival probabilities were markedly reduced among patients with high LAR values (all log-rank *p* < 0.001). These findings highlight the strong prognostic value of elevated LAR for both short- and intermediate-term survival outcomes in elderly SAP patients (Figure 3).

### 3.6 LAR-level and clinical outcomes

The associations between LAR levels and clinical outcomes are presented in Table 6. Compared with the low-LAR group, patients in the high-LAR group exhibited markedly higher in-hospital, 28-day, and 90-day mortality rates (all *p* < 0.001). Length of ICU stay was comparable between groups, whereas hospital stay was slightly shorter in the high-LAR group (*p* = 0.025).

**Table 6:** Relationship between LAR-level and clinical outcomes.

Variables	LAR		*p*
	Low (<0.79)	High (≥0.79)	
*N*	170	143	
In-hospital mortality, *n* (%)	31 (18.2)	63 (44.1)	<0.001
28-day mortality, *n* (%)	21 (12.4)	52 (36.4)	<0.001
90-day mortality, *n* (%)	28 (16.5)	63 (44.1)	<0.001
LOS ICU (days)	11 (5–20)	10 (4–18)	0.102
LOS hospital (days)	20 (10–23)	16 (8–27)	0.025

LAR, Lactate-to-albumin ratio; LOS, length of stay; ICU, intensive care unit stay.

### 3.7 Subgroup analysis

**Figure 4:**
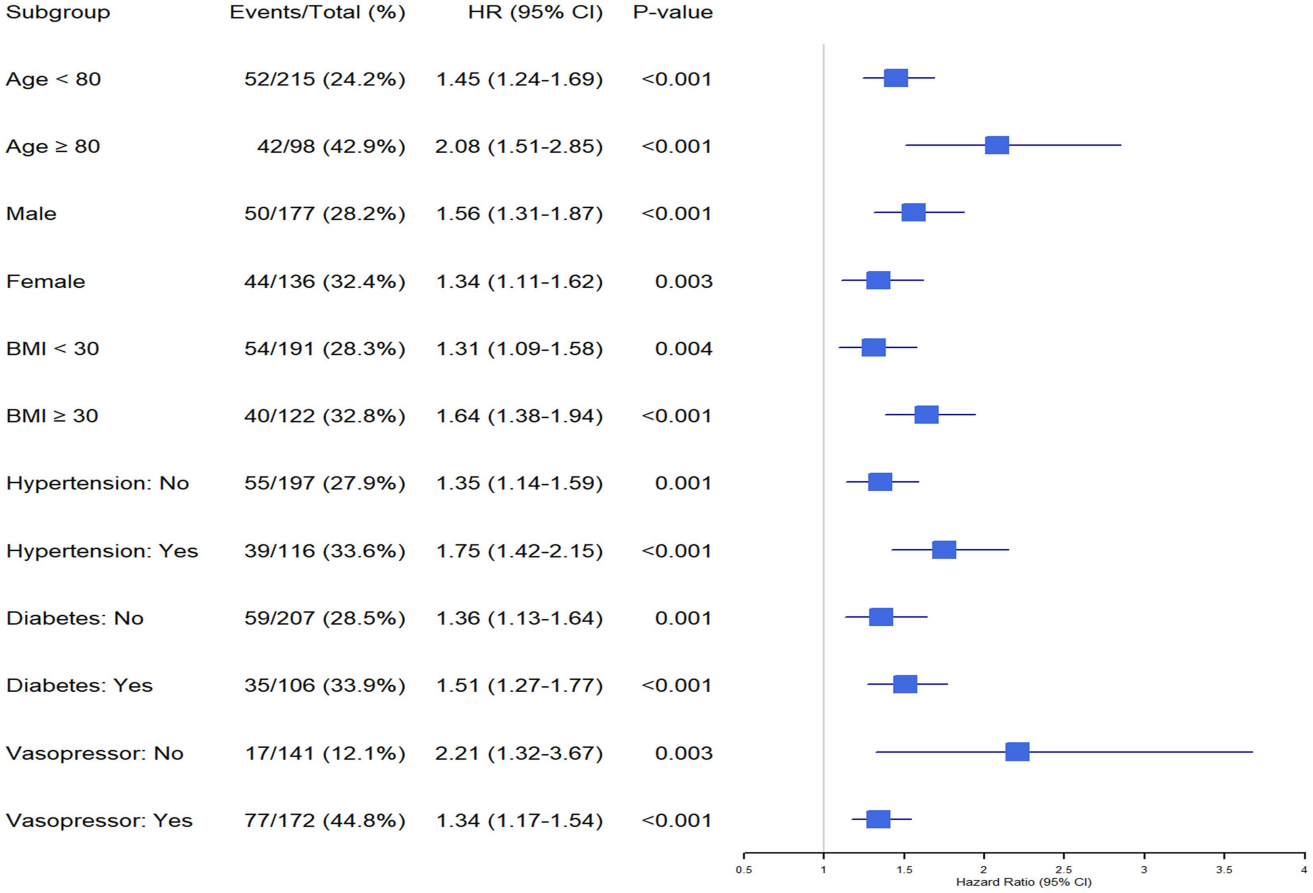
Forest plot for subgroup analysis of the relationship between in-hospital mortality and LAR. LAR, lactate-to-albumin ratio.

To assess the robustness of the association between LAR and hospital mortality, subgroup analyses were conducted according to age, sex, BMI, hypertension, diabetes mellitus, and vasopressor use. Across all subgroups, high LAR was consistently associated with an increased risk of mortality (Figure 4).

## 4 Discussion

Age is a well-established independent risk factor for mortality in AP (21). Although AP manifests similarly in younger and older patients, the elderly are more prone to severe complications, such as multisystem failure, particularly in acute necrotizing pancreatitis (22). To our knowledge, this is the first study to examine the prognostic value of the LAR in elderly patients with SAP. In this retrospective analysis, LAR emerged as an independent predictor of hospital mortality. The attenuation of the association after adjusting for SOFA suggests that disease severity may partly explain this relationship. Nevertheless, LAR remained independently associated with mortality, indicating additional prognostic value beyond established severity scores. ROC analysis showed that LAR (AUC = 0.700) outperformed albumin (AUC = 0.432) and was slightly superior to lactate (AUC = 0.688), though the latter difference was not statistically significant. While SOFA demonstrated superior clinical utility, LAR may still provide incremental value due to its simplicity and ease of use. Kaplan–Meier curves confirmed that patients with LAR ≥ 0.79 had significantly higher mortality than those with lower values.

In recent years, numerous attempts have been made to refine prognostic evaluation for AP. Multiple scoring frameworks, including the Ranson criteria, the Bedside Index for Severity in Acute Pancreatitis (BISAP), SOFA score, and APACHE II, are frequently utilized to classify risk and inform therapeutic decisions (23, 24). Nevertheless, these systems demonstrate only moderate predictive strength, with reported AUCs generally between 0.6 and 0.8 for outcomes such as sustained organ dysfunction, major complications, or mortality (25). Furthermore, most scoring tools require data collection over the first 48 h, which may postpone critical early management, as exemplified by the Ranson criteria that rely on several parameters assessed only after 48 h, such as hematocrit decrease, blood urea nitrogen increase, and hypocalcemia (26). To overcome these limitations, several inflammation-related indicators—including red cell distribution width, neutrophil-to-lymphocyte ratio (NLR), and C-reactive protein—have been examined as adjunctive markers (27). Among these, NLR has shown promising but inconsistent prognostic performance across studies. Junare *et al*. reported excellent discrimination of NLR for predicting ICU admission and mortality in AP, with AUC values exceeding 0.90 (28), whereas Mihoc *et al*. observed more modest performance, reporting an AUC of approximately 0.70 for mortality prediction in ICU-admitted patients with SAP (29). Yet, results across studies remain heterogeneous, restricting their translational applicability and complicating the establishment of uniform management standards.

Epidemiological studies have shown that the proportion of elderly patients among those with SAP is increasing, and this group often experiences longer hospital stays, higher complication rates, and poorer functional recovery after discharge (30). In SAP, patients ≥70 years face higher risks of organ failure and death (22), warranting closer monitoring. While some studies report worse outcomes in octogenarians, others find no difference from younger elderly patients (31), possibly due to variations in inflammatory response, comorbidities, and care strategies. In our study, age was independently associated with hospital mortality in elderly SAP patients, reinforcing its prognostic relevance. This highlights the need for careful monitoring and tailored treatment, especially for those with other health problems, to reduce this risk.

Lactate, generated during anaerobic metabolism, serves as a key indicator of insufficient tissue oxygenation and circulatory dysfunction (32). In the present cohort, lactate levels were markedly higher among non-survivors, and elevated concentrations remained an independent predictor of hospital death in elderly individuals with SAP. This observation echoes prior investigations. Using data from the MIMIC-IV database, Zeng *et al*. demonstrated that lactate independently predicted both early and late mortality in ICU patients with AP (13). Consistently, Shu *et al*. reported that an initial arterial lactate ≥ 4 mmol/L was strongly linked to worse outcomes in SAP, encompassing multiorgan failure, septic shock, and higher mortality (HR = 10, 95% CI: 3.7–27, *p* < 0.01) (33). Nevertheless, lactate interpretation warrants caution since impaired hepatic metabolism can lead to false elevation unrelated to tissue hypoperfusion, and agents such as salbutamol or metformin may increase its level through non-hypoxic mechanisms (34).

Albumin, a primary plasma protein produced by the liver, plays a vital role in maintaining homeostasis (35). Previous investigations have associated hypoalbuminemia with adverse outcomes, including increased hospital mortality in a large cohort of emergency patients in Ireland, as well as persistent organ failure in AP, as reported by Li et al. (36, 37) in Wuhan. In our cohort of elderly SAP patients, univariate Cox analysis did not reveal a significant link between low albumin levels and hospital mortality. The AUC of albumin in ROC analysis was below 0.5, which likely reflects the inverse relationship between albumin concentration and mortality risk. Because lower rather than higher values indicate worse prognosis, the discriminatory direction is reversed in conventional ROC analysis. This divergence may reflect the influence of factors such as nutritional status, fluid balance, and chronic inflammatory conditions, which can limit the prognostic reliability of albumin when considered in isolation (38).

The LAR has recently been recognized as a prognostic indicator in critically ill populations, including those with sepsis, heart failure, and respiratory failure, and has been shown to outperform lactate or albumin alone in predicting mortality (39). In patients with AP, Liu et al. demonstrated that elevated LAR independently predicted all-cause death (8), while Lu et al. reported its association with 30-day mortality in acute respiratory failure, with an AUC of 64.6%, slightly higher than that of the SOFA score (AUC 64.2%) (40). However, no prior studies have assessed its prognostic value in elderly SAP patients. Our study found that LAR remained a significant independent predictor of in-hospital mortality after adjustment for confounding factors, and its relationship with mortality appeared non-linear. The nonlinear association between LAR and mortality was further explored using a two-piecewise linear model, which allows for intuitive interpretation of risk across clinically relevant thresholds. Notably, the stronger association observed at lower LAR levels should be interpreted with caution. The relatively wide confidence interval and smaller subgroup size may indicate estimation instability, and the observed effect may be influenced by data distribution. Although HRs were expressed per 1-unit increase according to the model specification, changes of this magnitude are uncommon in daily practice. Smaller variations (e.g., 0.1 units) may therefore provide a more clinically realistic interpretation of risk.

These results support the potential utility of LAR as a straightforward biomarker for early identification of high-risk patients, which could aid timely clinical decision-making and risk stratification in this vulnerable cohort. From a clinical workflow perspective, the identified cutoff value (0.79) may be readily implemented in routine practice. As both lactate and albumin are routinely measured at ICU admission, LAR can be automatically calculated without additional burden. Patients with LAR ≥ 0.79 may be promptly identified as high-risk individuals, warranting intensified monitoring, closer hemodynamic assessment, and consideration of early escalation of supportive care.

This study has several limitations. First, as a single-center retrospective cohort, it limits causal inference between LAR and mortality in elderly SAP patients, thereby reducing the strength of evidence. Second, although we adjusted for multiple potential confounders, residual bias from unmeasured variables (e.g., nutritional status, disease etiology, and pre-hospital management) may persist. These unmeasured variables may have influenced both LAR levels and clinical outcomes. For instance, poor nutritional status may lead to lower albumin levels and worse prognosis, potentially resulting in an overestimation of the association between LAR and mortality. Similarly, variations in disease etiology or early treatment prior to ICU admission may also affect both exposure and outcome. Therefore, the observed associations should be interpreted with caution. Third, we only analyzed LAR values at ICU admission, which may overlook the prognostic impact of dynamic changes during hospitalization. Fourth, the sample size, while adequate for statistical analysis, may limit the generalizability of our findings to other populations or healthcare settings. In addition, although all variables were collected within 24 h of ICU admission, variability in the time from disease onset or initial hospital presentation to ICU admission may exist. Such differences could influence LAR levels and may affect its performance as an “early” prognostic marker. As this study focused on association analysis rather than the development of a formal prediction model, neither internal nor external validation was performed. Future multicenter, prospective studies with larger sample sizes, repeated LAR measurements, and inclusion of additional clinical variables are warranted to confirm and extend these results.

## 5 Conclusion

In our cohort, LAR was independently associated with in-hospital mortality in elderly patients with SAP. Given its simplicity and accessibility, LAR may serve as a complementary biomarker for early risk stratification, although it does not outperform established severity scoring systems.

## Data Availability

The raw data supporting the conclusions of this article will be made available by the authors, without undue reservation.
